# Enhanced Nanomagnetic Gene Transfection of Human Prenatal Cardiac Progenitor Cells and Adult Cardiomyocytes

**DOI:** 10.1371/journal.pone.0069812

**Published:** 2013-07-31

**Authors:** Mahendran Subramanian, Jenson Lim, Jon Dobson

**Affiliations:** 1 nanoTherics Limited, Keele University Science and Business Park, Newcastle under Lyme, Staffordshire, United Kingdom; 2 Institute of Microbiology and Infection, School of Biosciences, University of Birmingham, Edgbaston, Birmingham, United Kingdom; 3 J. Crayton Pruitt Family Department of Biomedical Engineering and Department of Material Science and Engineering, University of Florida, Gainesville, Florida, United States of America; 4 Institute for Cell Engineering and Regenerative Medicine, University of Florida Gainesville, Florida, United States of America; 5 Institute for Science and Technology in Medicine, Keele University, Stoke-on-Trent, Staffordshire, United Kingdom; Brandeis University, United States of America

## Abstract

Magnetic nanoparticle-based gene transfection has been shown to be an effective, non-viral technique for delivery of both plasmid DNA and siRNA into cells in culture. It has several advantages over other non-viral delivery techniques, such as short transfection times and high cell viability. These advantages have been demonstrated in a number of primary cells and cell lines. Here we report that oscillating magnet array-based nanomagnetic transfection significantly improves transfection efficiency in both human prenatal cardiac progenitor cells and adult cardiomyocytes when compared to static magnetofection, cationic lipid reagents and electroporation, while maintaining high cell viability. In addition, transfection of adult cardiomyocytes was improved further by seeding the cells onto Collagen I-coated plates, with transfection efficiencies of up to 49% compared to 24% with lipid reagents and 19% with electroporation. These results demonstrate that oscillating nanomagnetic transfection far outperforms other non-viral transfection techniques in these important cells.

## Introduction

The discovery, isolation and differentiation of human cardiac progenitor cells from the epicardium of the heart has given scientists and clinicians alike a tangible opportunity to investigate cardiovascular diseases as well as other issues concerning tissue regeneration [1], [2], [3], [4]. Cardiac progenitor cells, with their ability to differentiate into cardiomyocytes, fibroblasts and coronary vessels *in vitro,* are unique in their ability to replace damaged cardiac tissue in cardiovascular cell therapy [5], [6]. The isolation of primary adult human cardiomyocytes has made it possible to formulate *in vitro* models to understand the human heart and cardiac diseases [7], identify the different cardiomyocytes present [8], and study cardiomyocyte differentiation to address tissue regeneration [9]. However, despite these advances, successes in cardiovascular gene therapy still remains elusive and non-viral transfection of cardiomyocytes suffers from poor efficiency and relatively low cell viability.

There are more than 3,000 genetic disorders that arise as a result of single gene alterations. In the cardiovascular system disorders such as atrioventricular conduction delay, atrial septal defects, early valve calcification and endocardial cushion effect, all involve single gene alterations [10]. Understanding these mutations and their resulting disorders remains crucial in our search for a cure. While it may be possible to identify a disease-causing gene, delivery of genetic material into a cell to correct the defective gene remains a challenge. Currently, this is achieved mostly by using non-infective viruses, cationic lipid reagents and electroporation *in vitro* (Table 1). Most gene delivery approaches are not been widely applicable due to low transfection efficiency or the lack of suitable vectors, target specificity, or safety issues arising from translating the technique into humans [11].

**Table 1:** Review of methods used for transfecting cardiovascular system cells.

Cell type	Source	Transfection method	Transfection efficiency
Cardiovascular system cells	Chicken	Adenoviral vector	63% [38]
Cardiomyocytes	Rat	Adenoviral vector	90% [39]
Cardiomyocytes	Rat	Adenoviral vector	100% [40]
Cardiomyocytes	Rat	Adeno associated viral vector	90% [40]
Cardiomyocytes	Rat	Adeno associated viral vector	88.1% [41]
Cardiomyocytes	Rat	Adenoviral-polylysine vector	70% [42]
Cardiomyocytes	Rat	Retrovirus/Lentiviral vector	70–100% [40]
Cardiomyocytes	Rat	Calcium phosphate precipitate	1–2% [40]
Cardiomyocytes	Rat	Calcium phosphate precipitate	5–8% [41]
Cardiomyocytes	Rat	Calcium phosphate precipitation method	2–4% [43]
Cardiomyocytes	Rat	Calcium phosphate precipitation method	5–10% [54]
Cardiovascular system cells	Chicken	Electroporation	26% [38]
Cardiomyocytes	Rat	Electroporation	7.5% [41]
Cardiomyocytes	Rat	Electroporation	37% [40]
Cardiomyocytes	Rat	Nucleofection	4.8% [41]
Cardiomyocytes	Rat	Nucleofection	70% [40]
Cardiomyocytes	Rat	Lipofectin	1.6% [42]
Cardiomyocytes	Rat	Lipofectamine 2000	8.1% [41]
Cardiomyocytes	Rat	Fugene 6	3.3% [41]
Cardiomyocytes	Rat	Optical transfection	5% [40]
Cardiovascular system cells	Hamster	Hypothermic-cardioplegia method	77.3±9.0% [44]
Cardiomycytes	Rat	Pressure mediated transfection	48±5% [45]
Cardiomyocytes	Pig	Adenoviral vector	[46]
Cardiomyocytes	Rat	Adeno associated viral vector	[47]
Cardiac progenitor cells	Mice	Bicistronic lentiviral vector	[48]
Cardiovascular system cells	Dog	Hemagglutinating virus of Japan-liposome method	[49]
Cardiovascular system cells	Rat	Hemagglutinating virus of Japan–liposome method	[50]
Cardiomyocytes	Rat	Liposomes coated with uv inactivated sendai viral vector	[47]
Cardiomyocytes	Rat	Lipofectamine plus	[41]
Cardiomyocytes	Rat	Geneporter	[41]
Cardiomyocytes	Rat	Metafectene	[41]
Cardiomyocytes	Rat	Lipogen	[41]
Cardiovascular system cells	Rat	Ultrasound-targeted liposome microbubble destruction	[51]
Cardiovascular system cells	Dog	DNA-polymer coating	[52]
Cardiac progenitor cells	Human	The phiC31 integrase genomic modification system	[53]

In order to overcome these problems there is a critical need for an efficient, biocompatible and remotely controllable method of transfection. The use of magnetic nanoparticles (MNPs) has numerous applications in the field of biomedicine such as targeted drug delivery; diagnostics combined with therapeutics (i.e. magnetic resonance-guided stem cells labelled with MNPs for cell replacement therapy); MNP labelled-cell sorting; localised hyperthermia for the treatment of solid tumours, remote control of cell processes, and external magnetic field mediated gene delivery [12], [13], [14], [15].

Nanomagnetic transfection is a non-viral gene delivery technique that uses magnetic force acting on superparamagnetic nanoparticles (SPIONs) onto which plasmid DNA or siRNA is adsorbed. High gradient, rare earth magnets placed below the culture plate direct the MNP/DNA complexes into contact with cells and oscillating the magnet array induces endocytosis of the complex, after which the DNA is released into the cytoplasm [16], [17]. The advantages of magnetofection *in vitro* are: 1) low amounts of transfection complexes; 2) high cell viability; 3) high transfection efficiency; 4) little or no interference with cell proliferation and differentiation [18], [19], [20], [21], [22], [23], [24]. Although biocompatible MNPs have been developed specifically for magnetofection in various cell types with a stationary magnet array [19], [23], there have been no reports of the use of an oscillating magnet array during transfection of human prenatal cardiac progenitor cells and adult cardiomyocytes. Here we demonstrate the *in vitro* delivery of the reporter plasmid pEGFP-N1 using the oscillating magnet array and MNPs in human prenatal cardiac progenitor cells and adult cardiomyocytes and compare it to other non-viral transfection techniques.

## Materials and Methods

### Cells and Reagents

Human prenatal cardiac progenitor cells (Cambridge Bioscience, Cambridge, UK) and adult cardiomyocytes (Celprogen, California, USA) were purchased, cultured and maintained in standard tissue culture flasks containing horse serum supplemented Cardiac Cellutions medium (Cambridge Bioscience, Cambridge, UK) or in Extra Cellular Matrix (ECM) coated tissue culture flasks (Celprogen, California, USA) containing human cardiomyocytes cell culture complete media with serum and antibiotics (Celprogen, California, USA). The pEGFP-N1 (Clontech, California, USA), a 4.7 kb plasmid containing a CMV promoter and gene expressing enhanced Green Fluorescent Protein (eGFP) was used as a reporter for this study. The plasmid DNA was purified using EndoFree Plasmid Purification kit (Qiagen, Crawley, UK) and maintained at −80°C in endonuclease-free water (Sigma, Dorset, UK).

### Adhesion Assay

As human adult cardiomyocytes are a semi-adherent cell type, adhesion studies were performed. 96 well tissue culture plates (Corning, New York, USA) were coated with foetal bovine serum (FBS: 10, 5, 2.5, and 1.25%) (Lonza, Cologne, Germany), polyethyleneimine (PEI: 10, 5, 2.5, 1.25, 0.6 and 0.3 µg/ml) (Sigma, Dorset, UK), gelatin (0.1, 0.05 and 0.025% w/v) (Sigma, Dorset, UK) and rat tail collagen I (1, 0.5, 0.25 and 0.125 µg/ml) (Sigma, Dorset, UK), made up in phosphate buffer saline (PBS) and incubated overnight at 4°C. Following incubation the coated plates were washed once with PBS, 20,000 human adult cardiomyocytes were seeded onto coated wells and incubated overnight at 37°C with 5% CO_2_. 24 h post seeding, the wells were washed twice with PBS and stained with 2 mg/ml nuclear Hoechst-33342 stain (Sigma, Dorset, UK) at 1 µl per well. Cells adhered per field were imaged and phenotypes were recorded.

### Nanomagnetic Transfection

**Figure 1:**
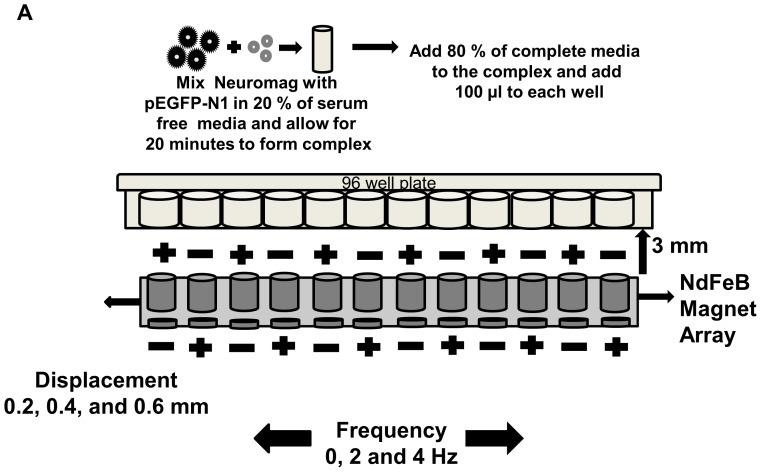
Oscillating nano-magnetofection 96 well experimental setup (a) Diagrammatic representation of a 96 well oscillating magnet array-based nanomagnetic transfection setup using NdFeB magnetic array.

10,000 progenitor cells and 20,000 cardiomyocytes per well were seeded into 96 well tissue culture plates previously coated with 0.8 µg/µl rat tail collagen I, and onto extra cellular matrix coated tissue culture treated 96 well plates (Celprogen, California, USA) in 100 µl complete growth media and incubated for 24 h at 37°C with 5% CO_2_. Prior to transfection, 0.05 or 0.1 µg of pEGFP-N1 formed complexes with 0.2 or 0.35 µl NeuroMag (nanoTherics, Keele, UK) for 15 minutes in serum-free growth media containing antibiotics (Celprogen, California, USA) (Figure 1). NeuroMag, a MNP transfection vector was used for its non-interference with proliferation and low cytotoxicity, as observed during transfection of oligodendrocyte progenitor cells [19] and neural precursor/stem cells [21], [23]. After complex formation, 80 µl of complete medium was added, mixed and transferred, drop-wise, onto the appropriate well(s) containing prenatal human cardiac progenitor cells or adult cardiomyocytes. Plates were placed on a 96 well oscillating NdFeB (Rare earth magnet, an alloy of neodymium, iron and boron) magnet array following the magnefect nano II system protocol (Figure 1). Following 30 minutes exposure to the oscillating magnetic array, the culture plate was removed from the system and incubated at 37°C with 5% CO_2_ for 48 h. Lipofectamine 2000 (Invitrogen, Paisley, UK) and Nucleofector 2b (Lonza, Cologne, Germany) were evaluated for comparison and transfections were performed according to the manufacturer’s instructions.

### Cell Viability Assay

CytoTox-ONE™ homogenous membrane integrity assay (Promega, Southampton, UK) provided the measure of released lactate dehydrogenase (LDH) through damaged membrane of dead cells and the assay was performed according to manufacturer’s instruction. Fluorescence was recorded at excitation wavelength 560 nm and emission wavelength 590 nm using a fluorescent plate reader (BioTek, Bedfordshire, UK). Viability data is expressed as a percentage using the following formula.

Percentage viability = 100– [100×(Experimental – Background fluorescence)/(Maximum LDH release – Background fluorescence)].

### Microscopy Analysis

48 h post transfection, cells were stained with 2 mg/ml Hoechst-33342 (nuclear) stain at 1 µl per well to ensure that only viable cells were counted while correlating GFP expressing cells. Both GFP expression and Hoechst fluorescence was observed using an epifluorescent microscope (Olympus IX51, Essex, UK). A semi-quantitative analysis of transfection efficiency was determined by counting the number of GFP-expressing cells versus the total number of Hoechst stained cells using the cell counting feature of ImageJ software (National Institute of Health, USA). Cell viability was determined by counting the number of cellTrace™ calcein red-orange-AM (Invitrogen, Paisley, UK) versus the total number of Hoechst stained cells and phase contrast microscopic image of cells using ImageJ software. Statistical significance was calculated using one-way analysis ANOVA with Bonferroni’s multiple comparison test (GraphPad Prism v6.01, California, USA).

## Results

### Oscillating Magnet Array-based Nanomagnetic Transfection of Human Cardiac Progenitor Cells

**Figure 2:**
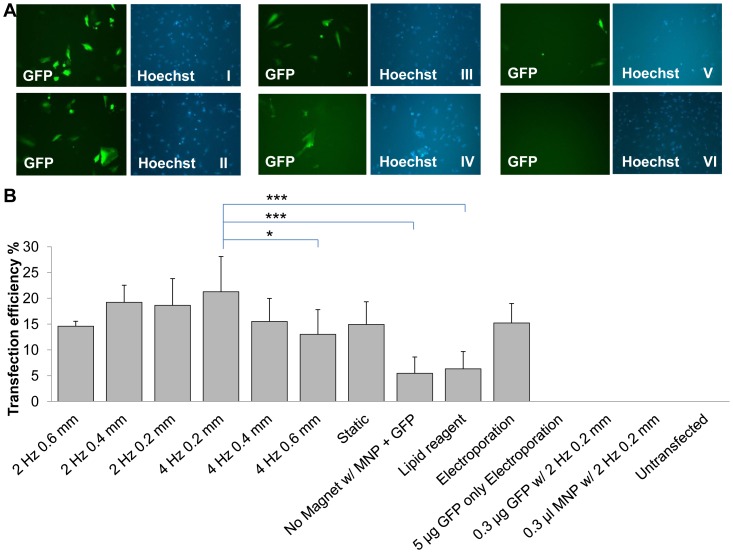
Comparison of prenatal human cardiac progenitor cells transfected using oscillating magnet array-based nanomagnetic transfection. (a) Fluorescent microscope images of prenatal human cardiac progenitor cells transfected with pEGFP-N1 representing each conditions, took 48 h post transfection. (I) GFP fluorescence and Hoechst image for 2 Hz 0.2 mm, (II) GFP fluorescence and Hoechst image for 4 Hz 0.2 mm, (III) GFP fluorescence and Hoechst image for static magnetofection™, (IV) GFP fluorescence and Hoechst image for electroporation (V) GFP fluorescence and Hoechst image for lipid based transfection reagent, (VI) GFP fluorescence and Hoechst image for un-transfected (b) Prenatal human cardiac progenitor cells were transfected with pEGFP-N1 as indicated, and scored using manual counting using ImageJ software, 48 h after transfection. Data shown are the media ± SD. n = 12 for 4 Hz 0.2 mm. n = 9 for 2 Hz 0.2 mm and static conditions. n = 6 for 4 Hz 0.4 mm, 4 Hz 0.6 mm, no magnet, electroporation and pEGFP-N1 only electroporation conditions. n = 3 for 2 Hz 0.4 mm, 2 Hz 0.6 mm, pEGFP-N1 only, Neuromag only, and un-transfected conditions. (*p<0.05, ***p<0.001 - Statistically significant).

In prenatal human cardiac progenitor cells approximately 18.6±5.2% transfection efficiency was observed when the complexes were subjected to an NdFeB magnet array oscillating at a frequency of 2 Hz with a displacement of 0.2 mm (Figure 2). Different oscillating frequencies (0, 2 and 4 Hz) and displacements (0.2, 0.4, 0.6 mm) were evaluated in order to ascertain their impact. Initial results were improved to 21.3±6.8% transfection efficiency using a magnet array oscillating at 4 Hz, with a 0.2 mm displacement. This appeared to be the optimum frequency/amplitude combination in this case and there was a decrease in transfection efficiency when compared to 2 Hz/0.2 mm (18.6±5.2%), 2 Hz/0.4 mm (19.2±3.3%, p>0.05) and 2 Hz/0.6 mm (14.6±0.9%, p>0.05), 4 Hz/0.4 mm (15.5±4.5%), and 4 Hz/0.6 mm (13.0±4.8%, *p<0.05). The 4 Hz/0.2 mm group also outperformed the no magnet control (5.5±3.2%, **p<0.001), static magnetofection (14.9±4.4%), cationic lipid-based transfection (6.3±3.4%, ***p<0.001), and electroporation (15.2±3.8%) (Figure 2). While there appears to be a suggestion of some systematic variation in transfection efficiency with displacement at 4 Hz, the relationship is not statistically significant across all three displacements and is not seen in the 2 Hz groups.

### Cell Adhesion Assay

**Figure 3:**
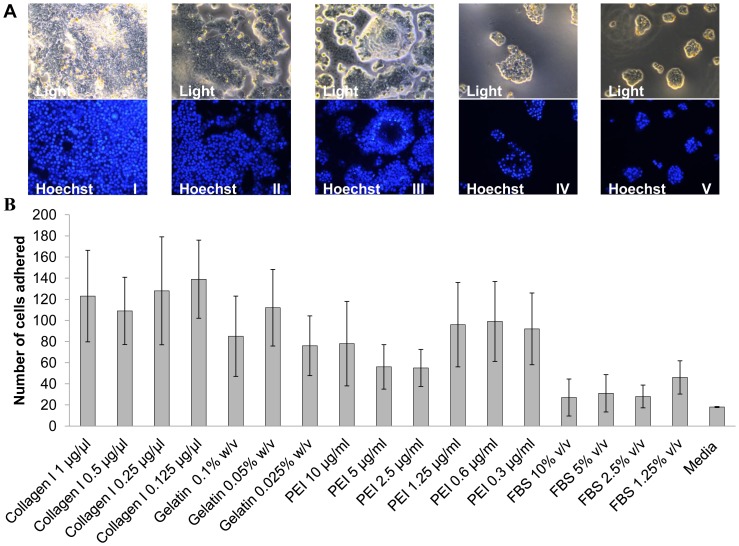
Adhesion studies in adult human cardiomyocytes. (a) Light and Fluorescent microscope images of adult human cardiomyocytes seeded onto different coatings, took after overnight incubation. (I) Light and Hoechst image for collagen 1 coating (0.125 µg/µl), (II) Light and Hoechst image for gelatine coating (0.05% w/v), (III) Light and Hoechst image for polyethyleneimine coating (0.6 µg/ml), (IV) Light and Hoechst image for foetal bovine serum coating (1.25%), and (V) Light and Hoechst image for no coating (b) adult human cardiomyocytes adhered per view as indicated, and scored using manual counting using ImageJ software, post 1× PBS wash after overnight incubation in the corresponding coatings. Data shown are the media ± SD. n = 4 for all the conditions.

In the adhesion studies, cells adhered better to wells coated with rat tail collagen I (0.125, 0.25, 0.5, 1 µg/ml), when compared to gelatin from bovine serum (0.05, 0.1, 0.025% W/v), polyethyleneimine (0.6, 1.25, 0.3, 10, 5, 2.5 µg/ml) and foetal bovine serum (1.25, 5, 2.5, 10% v/v). Morphologically, cells seeded onto collagen I-,gelatin-, FBS- and un- coated wells were normal in appearance, unlike cells seeded in polyethyleneimine-coated wells, which, though adhering, they were rounded, gathered in clusters with compacted nucleus and a high levels of cell debris were observed. With the latter case, cells were also observed to detach from the surface after washing (Figure 3). Cardiomyocytes being a semi-adherent cell type, collagen I was found to be a suitable coating to enhance adhesion in cardiomyocytes to aid adherent state transfection.

### Transfection of Human Adult Cardiomyocytes

**Figure 4:**
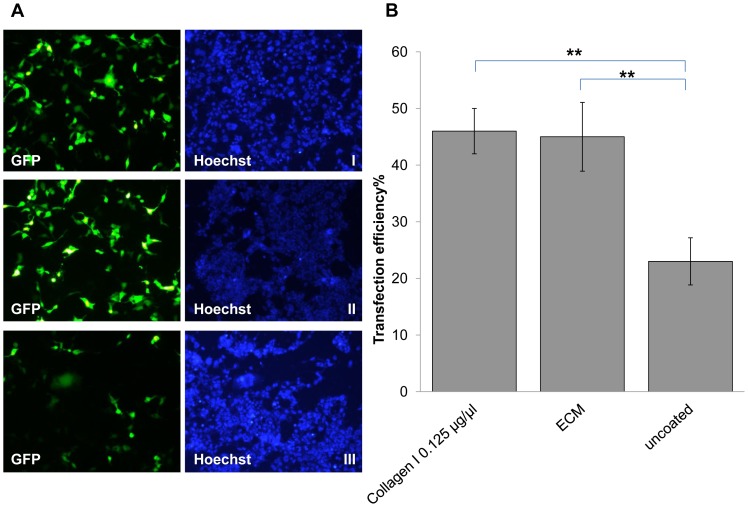
Comparison of oscillating magnet array-based nanomagnetic transfection between adult human cardiomyocytes seeded onto rat tail collagen 1 coated and ECM coated plates. (a) Fluorescent microscope images of adult human cardiomyocytes transfected with pEGFP-N1 representing each conditions, took 48 hrs post transfection. (I) GFP fluorescence and Hoechst image for 2 Hz 0.2 mm in collagen 1 coated (0.125 µg/µl) plate, (II) GFP fluorescence and Hoechst image for 2 Hz 0.2 mm in ECM coated plate, and (III) GFP fluorescence and Hoechst image for 2 Hz 0.2 mm in un-coated plate (b) adult human cardiomyocytes were transfected with pEGFP-N1 as indicated, and scored using manual counting using Image J software, 48 h after transfection. Data shown are the media ± SD. n = 3 for all the conditions. (**p<0.01- Statistically significant).

Human adult cardiomyocytes were transfected using an oscillating magnet array on the magnefect-nanoII system at a frequency of 2 Hz with a 0.2 mm displacement. No difference in transfection efficiency between the cells coated with Collagen I (46.0±4.0%) and ECM (44.8±6.1%, p>0.05) coated plates was observed. However, cells seeded onto uncoated plates exhibited low transfection efficiency (22.7±4.2%, **p<0.01) compared to the other conditions as shown in Figure 4. The results demonstrate that oscillating magnetofection can transfect adherent cells efficiently when compared to semi-adherent cells. Following this we evaluated the oscillating nanomagnetic transfection technique against another adherent state transfection and a suspension state transfection methods i.e. lipid- and electroporation-based technologies.

**Figure 5:**
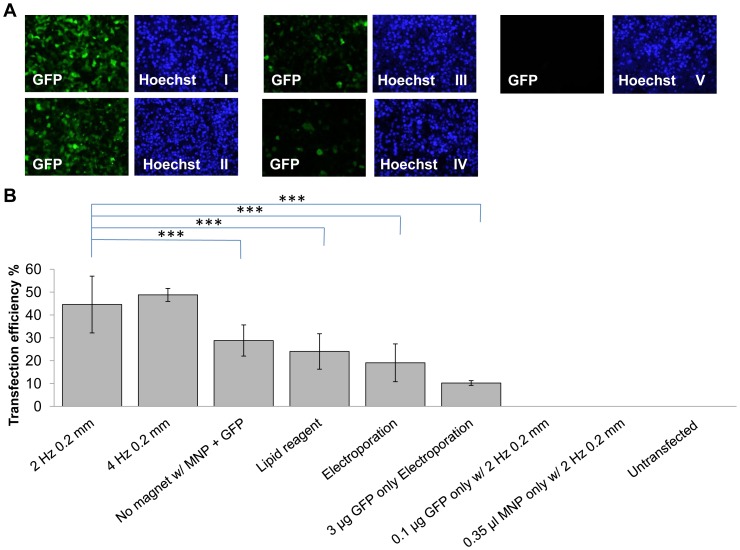
Comparison of oscillating magnet array-based nanomagnetic transfection over lipid based transfection and electroporation in adult human cardiomyocytes. (a) Fluorescent microscope images of adult human cardiomyocytes transfected with pEGFP-N1 representing each conditions, took 48 h post transfection. (I) GFP fluorescence and Hoechst image for oscillating magnet array-based nanomagnetic transfection performed at 2 Hz 0.2 mm, (II) GFP fluorescence and Hoechst image for oscillating magnet array-based nanomagnetic transfection performed at 4 Hz 0.2 mm (III) GFP fluorescence and Hoechst image for lipid based transfection reagent, (IV) GFP fluorescence and Hoechst image for electroporation and (V) GFP fluorescence and Hoechst image for untransfected (b) adult human cardiomyocytes were transfected with pEGFP-N1 as indicated, and scored using manual counting using Image J software, 48 h after transfection. Data shown are the media ± SD. n = 18 for oscillating magnet array-based nanomagnetic transfection at 2 Hz 0.2 mm. n = 12 for lipid based transfection reagent. n = 6 for oscillating magnet array-based nanomagnetic transfection at 4 Hz 0.2 mm, electroporation and pEGFP-N1 only electroporation conditions. n = 9 for no magnet. n = 3 for pEGFP-N1 only, Neuromag only, and un-transfected conditions. (***p<0.001- Statistically significant).

Cardiomyocytes transfected using the oscillating magnet array on the magnefect system at 4 Hz frequency/0.2 mm displacement (TE = 48.8±2.8%) and at 2 Hz 0.2 mm (TE = 44.6±12.4%) outperformed both a commonly used cationic lipid (TE = 24.0±7.8%, ***p<0.001) and electroporation (19.1±8.3%, ***p<0.001). At 4 Hz 0.2 mm (48.8±2.8%) and 2 Hz 0.2 mm (44.6±12.4%)) conditions cardiomyocytes showed significant increase in transfection efficiency when compared to no magnet control (28.8±6.8%, p<0.001) as shown in Figure 5. Overall these results demonstrate successful and efficient delivery of gene into cardiomyocytes using an oscillating magnet array and MNPs.

### Cell Viability

Cell viability based on the CytoTox-ONE™ homogenous membrane integrity assay for human prenatal cardiac progenitor cells was: static magnetofection (90.8±10.4%); 2 Hz/0.2 mm (84.4±13.9%); and 4 Hz/0.2 mm (76.3±25.0%); n = 3 for all groups. The cell viability based on cellTrace™ calcein red-orange AM staining for human adult cardiomyocytes was: no magnet (92.8±2.3%); 2 Hz/0.2 mm (89.7±5.6%); and 4 Hz/0.2 mm (69.5±8.4%); n = 9 for all groups. Overall the results demonstrate that MNP-based gene delivery appears to have minimal impact on the viability of cardiac progenitor cells and cardiomyocytes at static and low frequencies, however, a decrease in viability with the increasing frequency was observed.

## Discussion

Recent studies have demonstrated that, in some cell types, the use of oscillating magnet arrays on the magnefect system may enhance nanomagnetic gene transfection in comparison to static magnetofection [25], [26], [27]. It appears that cellular uptake of complexes during MNP mediated transfection is through endocytosis [28], especially through caveolae-mediated endocytosis in oscillating nanomagnetic gene transfection [16]. However, there may be effects due to oscillations that occur after the complex has been internalized that facilitate transfection.

Both cardiac progenitor cells and adult cardiomyocytes are important cell types for studies of the genetic basis of heart disorders such as atrioventricular conduction delay, atrial septal defects, early valve calcification and endocardial cushion effect. In order to understand these diseases and develop treatments, it is necessary to develop efficient and safe gene transfection techniques that also have the potential to be translated to the clinic. The oscillating nanomagnetic transfection technology demonstrated here outperforms other non-viral transfection techniques and, as MNPs are in clinical use as MRI contrast agents, has the potential to be translated into clinical application, such as transplantation of genetically altered cells.

In this study, we have shown that it is possible to optimize oscillating nanomagnetic transfection by investigating a range of frequencies and amplitudes. Similar transfection studies using oscillating nanomagnetic transfection on mouse embryonic fibroblasts, human umbilical vein endothelial cells [17], rat oligodendrocyte precursor cells [19], rat astrocytes [27], MG63 and NCI-H292 cells [29], [25] demonstrates the versatility of the frequency-displacement based-nanomagnetic transfection technology as well efficiency over a range of primary, differentiated, undifferentiated cells and cell lines. The reason behind frequency-displacement dependent transfection remains elusive; however, increased cytosolic Ca*^2+^* transients and enhanced Ca*^2+^* oscillations have been observed with increasing pacing frequency in adult mouse myocytes [30]. Similarly, shear stress induced cytosolic Ca*^2+^* transients are demonstrated in different cell types [31], [32]. The frequency-displacement dependent transfection we observe may depend on the cytosolic Ca*^2+^* oscillations, which is reported to interfere with the transfection and gene expression of cells [33], [34].

Moreover oscillating nanomagnetic transfection being an adherent cell transfection technique, we have also demonstrated that transfection efficiency can be enhanced by coating the wells with collagen. It is likely that increased efficiency in cells seeded onto rat tail collagen I was due to efficient adherence as collagen type-I is one of the fibrous proteins within extracellular matrix and, being a structural protein, it is highly biocompatible. In the cardiomyocytes, this led to increased adhesion and improved transfection efficiency with the oscillating system, while PEI and foetal bovine serum coating had a detrimental effect on the cells. This study represents a promising stepping stone towards a valuable research tool for cardiac gene therapy.

Oscillating nanomagnetic transfection optimisation may also be influenced by the properties of the MNPs used. While it is true that increases in the magnetic content (volume of magnetic material) of the particles will increase the magnetic force, generally the particles used for these studies are iron oxide/polymer composite particles. Increasing the hydrodynamic diameter is not always associated with a proportional increase in magnetic iron oxide as the polymer represents the bulk of the particle’s volume. Previous studies on MNP-mediated gene delivery has shown that 100 nm MNPs (hydrodynamic diameter) were endocytosed through caveolae-mediated endocytosis [16]; 200 nm MNPs through clathrin- or caveolae-mediated endocytosis [35]; and 300 nm MNPs through macropinocytosis and clathrin-mediated endocytosis [36]. Receptor mediated endocytosis has been involved either totally or partially in these demonstrated entry mechanisms of different sized nanoparticles. A diameter of 50–60 nm is considered to be suitable for optimal receptor mediated endocytosis [37], however the hydrodynamic diameter of Neuromag used in this study ranges between 140–200 nm [27]. Clearly the magnetic force on the particles, while important, is only one parameter to be considered as different entry mechanisms may be associated with increased or decreased transfection efficiency. Still, MNP-mediated gene delivery depends to some degree on the attractive force exerted by the magnet array on the magnetic particles [25]. The attractive force is directly proportional to the volume of magnetic material in the particle, magnetic field strength/gradient of the magnet array and magnetic properties (susceptibility) of the particle [18]. Superparamagnetic nanoparticles have high magnetic susceptibility but are limited in size to diameters of roughly 30 nm or less for iron oxides (though this is somewhat dependent on aspect ratios as magnetite has strong shape anisotropy). Suitable biocompatible polymer coating/matrices are also important for enhancing transfection, alongside size and magnetic properties of the nanoparticles [28].

## References

[1] Cai CL, Martin JC, Sun Y, Cui L, Wang L, et al (2008) A myocardial lineage derives from Tbx 18 epicardial cells. Nature 454: 104–108. 18480752 10.1038/nature06969 PMC5540369

[2] Smart N, Risebro CA, Melville AA, Mozes K, Schwartz RJ, et al (2007) Thymosin 4 induces adult epicardial progenitor mobilization and neovascularization. Nature 445: 177–182. 17108969 10.1038/nature05383

[3] Winter EM, Grauss RW, Hogers B, van Tuyn J, van der Geest R, et al (2007) Preservation of left ventricular function and attenuation of remodelling after transplantation of human epicardium-derived cells into the infarcted mouse heart. Circulation 116: 917–927. 17684151 10.1161/CIRCULATIONAHA.106.668178

[4] Zhou B, Ma Q, Rajagopal S, Wu SM, Domian I, et al (2008) Epicardial progenitors contribute to the cardiomyocyte lineage in the developing heart. Nature 454: 109–113. 18568026 10.1038/nature07060 PMC2574791

[5] Bearzi C, Rota M, Hosoda T, Tillmanns J, Nascimbene A, et al (2007) Human Cardiac Stem Cells. PNAS 104: 14068–14073. 17709737 10.1073/pnas.0706760104 PMC1955818

[6] Bollini S, Smart N, Riley PR (2011) Resident cardiac progenitor cells: at the heart of regeneration. Journal of Molecular and Cellular Cardiology 50: 296–303. 20643135 10.1016/j.yjmcc.2010.07.006

[7] Del Monte F, Harding SE, Schmidt U, Matsui T, Kang ZB, et al (1999) Restoration of contractile function in isolated cardiomyocytes from failing human hearts by gene transfer of SERCA2a. Circulation 100: 2308–2311. 10587333 10.1161/01.cir.100.23.2308 PMC1249502

[8] Bird SD, Doevendans PA, van Rooijen MA, Brutel de la Riviere A, Hassink RJ, et al (2003) The human adult cardiomyocyte phenotype. Cardiovascular Research 58: 423–434. 12757876 10.1016/s0008-6363(03)00253-0

[9] Perán M, Marchal JA, López E, Jiménez-Navarro M, Boulaiz H, et al (2010) Human cardiac tissue induces transdifferentiation of adult stem cells towards cardiomyocytes. Cytotherapy 12: 332–337. 20230311 10.3109/14653240903548202

[10] Richards AA, Garg V (2010) Genetics of congenital heart disease. Current Cardiology Reviews 6: 91–97. 21532774 10.2174/157340310791162703 PMC2892081

[11] Katz MG, Swain JD, White JD, Low D, Stedman H, et al (2010) Cardiac gene therapy: optimization of gene delivery techniques in vivo. Human Gene Therapy 21: 371–380. 19947886 10.1089/hum.2009.164 PMC2865214

[12] Dobson J (2008) Nanomagnetic actuation: remote control of cells. Nature Nanotechnology 3: 139–143. 10.1038/nnano.2008.39 18654485

[13] Pankhurst QA, Connolly J, Jones SK, Dobson J (2003) Applications of magnetic nanoparticles in biomedicine. Journal of physics D: Applied physics 36: R167–R181.

[14] Pankhurst QA, Thanh NKT, Jones SK, Dobson J (2009) Progress in applications of magnetic nanoparticles in biomedicine. Journal of Physics D: Applied Physics 42: 224001 (15pp)..

[15] Shubayev VI, Pisanic TRII, Jin S (2009) Magnetic nanoparticles for theragnostics. Advanced Drug Delivery Reviews 61: 467–477. 19389434 10.1016/j.addr.2009.03.007 PMC2700776

[16] Lim J, Clements MA, Dobson J (2012) Delivery of Short Interfering Ribonucleic Acid-Complexed Magnetic Nanoparticles in an Oscillating Field Occurs via Caveolae-Mediated Endocytosis. PLoS ONE 7(12): e51350 doi:10.1371/journal.pone.0051350 23236481 10.1371/journal.pone.0051350 PMC3517400

[17] Lim J, Dobson J (2012) Improved transfection efficiency of HUVEC and MEF cells using DNA-complexes with magnetic nanoparticles in an oscillating magnetic field. Journal of Genetics 91: 223–227. 22942095 10.1007/s12041-012-0164-4

[18] Dobson J (2006) Gene therapy progress and prospects: magnetic nanoparticle-based gene delivery. Nature Gene Therapy 13: 283–287. 10.1038/sj.gt.3302720 16462855

[19] Jenkins SI, Pickard MR, Granger N, Chari DM (2011) Magnetic nanoparticle- mediated gene transfer to oligodendrocyte precursor cell transplant populations is enhanced by magnetofection strategies. ACS Nano 5: 6527–6538. 21721568 10.1021/nn2018717

[20] Mykhaylyk O, Vlaskou D, Tresilwised N, Pithayanukul P, Moller W, et al (2007) Magnetic nanoparticle formulation for DNA and siRNA delivery. Journal of Magnetism and Magnetic materials 311: 275–281.

[21] Pickard MR, Barraud P, Chari DM (2011) The transfection of multipotent neural precursor/stem cell transplant populations with magnetic nanoparticles. Biomaterials 32: 2274–2284. 21193228 10.1016/j.biomaterials.2010.12.007

[22] Plank C, Rosenecker J (2009) Magnetofection: The use of magnetic nanoparticles for nucleic acid delivery. Cold Spring Harbour Protocol Doi:10.1101/pdb.prot5230. 10.1101/pdb.prot5230 20147188

[23] Sapet C, Laurent N, de Chevigny A, Le Gourrierec L, Bertosio E, et al (2011) High transfection efficiency of neural stem cells with magnetofection. Biotechniques 50: 187–189. 21486240 10.2144/000113628

[24] Scherer F, Anton M, Schillinger U, Henke J, Bergemann C, et al (2002) Magnetofection: enhancing and targeting gene delivery by magnetic force in vitro and in vivo. Nature Gene Therapy 9: 102–109. 10.1038/sj.gt.3301624 11857068

[25] Fouriki A, Farrow N, Clements MA, Dobson J (2010) Evaluation of the magnetic field requirements for nanomagnetic gene transfection. Nano Reviews doi: 10.3402/nano.v1i0.5167. 10.3402/nano.v1i0.5167 PMC3215215 22110859

[26] McBain SC, Grienenbach U, Xenariou S, Keramane A, Batich CD, et al (2008) Magnetic nanoparticles as gene delivery agents: enhanced transfection in the presence of oscillating magnet arrays. Nanotechnology 19: 405102. 21832609 10.1088/0957-4484/19/40/405102

[27] Pickard MR, Chari DM (2010) Enhancement of magnetic nanoparticle-mediated gene transfer to astrocytes by ‘magnetofection’: effects of static and oscillating fields. Nanomedicine 5: 217–232. 20148634 10.2217/nnm.09.109

[28] Kami D, Takeda S, Itakura Y, Gojo S, Watanabe M, et al (2011) Application of Magnetic Nanoparticles to Gene Delivery. International Journal of Molecular Sciences 12: 3705–3722. 21747701 10.3390/ijms12063705 PMC3131585

[29] Fouriki A, Clements MA, Farrow N, Dobson J (2013) Efficient transfection of MG63 osteoblasts using magnetic nanoparticles and oscillating magnetic fields. Tissue engineering & regenerative medicine. In press (DOI: 10.1002/term.1508). 10.1002/term.1508 22499386

[30] Lim CC, Apstein CS, Colucci WS, Liao R (2000) Impaired cell shortening and relengthening with increased pacing frequency are intrinsic to the senescent mouse cardiomyocyte. Journal of Molecular and Cellular Cardiology 32: 2075–2082. 11040110 10.1006/jmcc.2000.1239

[31] Schwarz G, Callewaert G, Droogmans G, Nilius B (1992) Shear stress-induced calcium transients in endothelial cells from human umbilical cord veins. The Journal of Physiology 458: 527–538. 1338792 10.1113/jphysiol.1992.sp019432 PMC1175170

[32] Wei F, Shi X, Chen J, Zhou L (2012) Fluid shear stress-induced cytosolic calcium signalling and degranulation dynamics in mast cells. Cell Biology International Reports 19: 45–51.

[33] Dolmetsch RE, Xu K, Lewis RS (1998) Calcium oscillations increase the efficiency and specificity of gene expression. Nature 392: 933–936. 9582075 10.1038/31960

[34] Preuss AK, Connor JA, Vogel H (2000) Transient transfection induces different intracellular calcium signaling in CHO K1 versus HEK 293 cells. Cytotechnology 33: 139–145. 19002821 10.1023/A:1008150402616 PMC3466731

[35] Huth S, Lausier J, Gersting SW, Rudolph C, Plank C, et al (2004) Insights into the mechanism of magnetofection using PEI-based magnetofectins for gene transfer. The Journal of Gene Medicine 6: 923–936. 15293351 10.1002/jgm.577

[36] Pickard MR, Jenkins SI, Koller CJ, Furness DN, Chari DM (2011) Magnetic Nanoparticle Labeling of Astrocytes Derived for Neural Transplantation. Tissue Engineering Part C Methods 17: 89–99. 20666601 10.1089/ten.TEC.2010.0170

[37] Zhang S, Li J, Lykotrafitis G, Bao G, Suresh S (2009) Size-Dependent Endocytosis of Nanoparticles. Advanced Materials 21: 419–424. 19606281 10.1002/adma.200801393 PMC2709876

[38] Harrison RL, Byrne BJ, Tung L (1998) Electroporation-mediated gene transfer in cardiac tissue. FEBS Letters 435: 1–5. 9755847 10.1016/s0014-5793(98)00987-9

[39] Frank KF, Bölck B, Ding Z, Krause D, Hattebuhr N, et al (2005) Overexpression of sorcin enhances cardiac contractility in vivo and in vitro. Journal of Molecular and Cellular cardiology 38: 607–615. 15808837 10.1016/j.yjmcc.2005.01.011

[40] Louch WE, Sheehan KA, Wolska BM (2011) Methods in cardiomyocyte isolation, culture, and gene transfer. Journal of Molecular and Cellular Cardiology 51 (2011) 288–298. 10.1016/j.yjmcc.2011.06.012 PMC3164875 21723873

[41] Djurovic S, Iverson N, Jeansson S, Hoover F Christenson G (2004) Comparison of nonviral transfection and adeno-associated viral transduction on cardiomyocytes. Molecular Biotechnology 28: 21–31. 15456960 10.1385/MB:28:1:21

[42] Kohout TA, O’Brian JJ, Gaa ST, Lederer WJ, Rogers TB (1996) Novel adenovirus component system that transfects cultured cardiac cells with high efficiency. Circulation Research 78: 971–977. 8635247 10.1161/01.res.78.6.971

[43] Takemoto M, Node K, Nakagami H, Liao Y, Grimm M, et al (2001) Statins as antioxidant therapy for preventing cardiac myocyte hypertrophy. The Journal of Clinical Investigation 108: 1429–1437. 11714734 10.1172/JCI13350 PMC209420

[44] Ikeda Y, Gu Y, Iwanaga Y, Hoshijima M, Oh SS, et al (2002) Restoration of deficient membrane proteins in the cardiomyopathic hamster by in vivo cardiac gene transfer. Circulation 105: 502–508. 11815435 10.1161/hc0402.102953

[45] Poston RS, Mann MJ, Hoyt EG, Ennen M, Dzau VJ, et al (1999) Antisense oligodeoxynucleotides prevent acute cardiac allograft rejection via a novel, nontoxic, highly efficient transfection method. Transplantation 68: 825–832. 10515383 10.1097/00007890-199909270-00015

[46] Shi W, Schmarkey LS, Jiang R, Bone CC, Condit ME, et al (2012) Ischemia-repurfusion increases transfection efficiency of intracoronary adenovirus type 5 in pig heart in situ. Human Gene Therapy Methods 23: 1–9. 22816318 10.1089/hgtb.2012.048 PMC4015071

[47] Kawaguchi H, Shin WS, Wang Y, Inukai M, Kato M, et al (1997) In vivo gene transfection of human endothelial nitric oxide synthase in cardiomyocytes causes apoptosis-like cell death. Circulation 95: 2441–2447. 9170408 10.1161/01.cir.95.10.2441

[48] Fischer KM, Cottage CT, Wu W, Din S, Gude NA, et al (2009) Enhancement of myocardial regeneration through genetic engineering of cardiac progenitor cells expressing Pim-1 Kinase. Circulation 120: 2077–2087. 19901187 10.1161/CIRCULATIONAHA.109.884403 PMC2787902

[49] Ahmet I, Sawa Y, Iwata K, Matsuda H (2002) Gene transfection of hepatocyte growth factor attenuates cardiac remodelling in the canine heart: A novel gene therapy for cardiomyopathy. The Journal of Thoracic and Cardiovascular Surgery 124: 957–963. 12407379 10.1067/mtc.2002.126655

[50] Jayakumar J, Suzuki K, Khan M, Smolenski RT, Farrell A, et al.. (2000) Gene therapy for myocardial protection. Transfection of donor hearts with heat shock protein 70 gene protects cardiac function against ischemia-reperfusion injury. Circulation 102: Iii 302-Iii 306. 10.1161/01.cir.102.suppl_3.iii-302 11082405

[51] Chen S, Shohet RV, Bekeredjian R, Frenkel P, Grayburn PA (2003) Optimization of ultrasound parameters for cardiac gene delivery of adenoviral or plasmid deoxyribonucleic acid by ultrasound-targeted microbubble destruction. Journal of the American College of Cardiology 42: 301–308. 12875768 10.1016/s0735-1097(03)00627-2

[52] Labhasetwar V, Bonadio J, Goldstein S, Chen W, Levy RJ (1998) A DNA controlled-release coating for gene transfer: Transfection in skeletal and cardiac muscle. Journal of Pharmaceutical Sciences 87: 1347–1350. 9811488 10.1021/js980077+

[53] Lan F, Liu J, Narsinh KH, Hu S, Han L, et al (2012) Safe genetic modification of cardiac stem cells using a site-specific integration technique. Circulation 126: s20–s28. 22965984 10.1161/CIRCULATIONAHA.111.084913 PMC3481839

[54] Barger PM, Brandt JM, Leone TC, Weinheimer CJ, Kelly DP (2000) Deactivation of peroxisome proliferator–activated receptor-α during cardiac hypertrophic growth. The Journal of Clinical Investigation 105: 1723–1730. 10862787 10.1172/JCI9056 PMC378509

